# Teleguidance-based remote navigation assistance for visually impaired and blind people—usability and user experience

**DOI:** 10.1007/s10055-021-00536-z

**Published:** 2021-05-24

**Authors:** Babar Chaudary, Sami Pohjolainen, Saima Aziz, Leena Arhippainen, Petri Pulli

**Affiliations:** 1 Faculty of Information Technology and Electrical Engineering, OASIS Research Unit, University of Oulu, PO Box 3000, 90014 Oulu, Finland; 2 Lahore, Punjab, Pakistan; 3 Faculty of Information Technology and Electrical Engineering, INTERACT Research Unit, INTERACT Research Unit, University of Oulu, PO Box 8000, 90014 Oulu, Finland

**Keywords:** Visual impairment, Navigation assistance, Remote assistance, Teleguidance, Haptic, Voice-based communication

## Abstract

This paper reports the development of a specialized teleguidance-based navigation assistance system for the blind and the visually impaired. We present findings from a usability and user experience study conducted with 11 blind and visually impaired participants and a sighted caretaker. Participants sent live video feed of their field of view to the remote caretaker’s terminal from a smartphone camera attached to their chest. The caretaker used this video feed to guide them through indoor and outdoor navigation scenarios using a combination of haptic and voice-based communication. Haptic feedback was provided through vibrating actuators installed in the grip of a Smart Cane. Two haptic methods for directional guidance were tested: (1) two vibrating actuators to guide left and right movement and (2) a single vibrating actuator with differentiating vibration patterns for the same purpose. Users feedback was collected using a meCUE 2.0 standardized questionnaire, interviews, and group discussions. Participants’ perceptions toward the proposed navigation assistance system were positive. Blind participants preferred vibrational guidance with two actuators, while partially blind participants preferred the single actuator method. Familiarity with cane use and age were important factors in the choice of haptic methods by both blind and partially blind users. It was found that smartphone camera provided sufficient field of view for remote assistance; position and angle are nonetheless important considerations. Ultimately, more research is needed to confirm our preliminary findings. We also present an expanded evaluation model developed to carry out further research on assistive systems.

## Introduction

People with visual impairments face major challenges in their daily lives. They tend to suffer from adverse effects on performance in many situations (Bhatlawande et al. 2014). According to projections, globally there were around 38.5 million blind people and 237.1 million people with moderate or severe visual impairment in 2020. These numbers are predicted to increase almost threefold to 114.6 million and 587.6 million, respectively, by 2050. As a result, a significantly higher number of people will require assistive technologies (AT) in the future (Bourne et al. 2017). In this paper, the acronym VIP is used to indicate blind or visually impaired people. Improving the mobility and navigation of VIPs has been and continues to be one of the primary areas of research and development focus when it comes to AT. This is reflected in societal goals that aim to help VIPs remain independent and integrated into society. This, in turn, will improve their quality of life (Calder 2009; Bohwmick and Hazarika 2017).

In general, adoption of AT has been a significant barrier among people with disabilities, and discontinuance rates tend to be high. Although some VIPs do use the latest technology, research has highlighted many reasons why acceptance remains low (Riemer-Reiss and Wacker 2000; Roentgen et al. 2008; Paajala and Keränen 2015; Gori et al. 2016). Developing products for VIPs is therefore an ongoing challenge. When evaluating new assistive technologies, developers tend to report very positive results from end users (Bhatlawande et al. 2014). However, according to Roentgen et al. (2008), many of these products fail to meet the needs of VIPs. Calder (2009) and Riemer-Reiss and Wacker (2000) have outlined the many difficulties inherent in designing AT products. First, it is difficult to have a lasting effect in the market, as it is hard to match the user requirements of an evolving ecosystem of products; VIPs often have multiple disabilities that demand different products. Second, solutions that use a variety of technologies—including different kinds of sensors, networks, user interfaces, and so on—add to the challenge of developing AT with longevity. Furthermore, rapid advances in technology can make even proven solutions obsolete or undesirable. A good example of this is the arrival of affordable smartphones and the availability of mobile applications (Calder 2009; Riermer-Reiss and Wacker 2002; Roentgen et al. 2008; Islam et al. 2019).

This paper reports the development of a specialized teleguidance-based navigation assistance system for blind and visually impaired users and preliminary findings from an experiment conducted in Pakistan. In the following section, the background for this study is presented. Next, the research methodology and the target system are described. The fourth section then presents our findings based on the usability test, interviews, observations, and the meCUE 2.0 questionnaire data. We then submit an extended evaluation model for consideration. This was developed with further research in mind and can be used to carry out evaluations for similar systems. Finally, we discuss our findings, reflect on their limitations, and consider possible future work before concluding the paper.

## Research context

VIPs use two kinds of assistance: primary and secondary. Primary assistance includes widely adopted assistances such as white canes and guide dogs. Secondary assistance consists of a variety of products in two broader categories. The first is electronic travel aids (ETAs), which sense a nearfield environment for mobility assistance. The second is orientation and navigation systems (ONS), which assist in reaching far field (Loomis et al. 2007; Cardillo and Caddemi, 2019). ETAs aim to improve obstacle avoidance by in a way extending the length of the white cane. However, they do not help with object localization or more complex navigation tasks; technologies used to enhance these ETAs often include sonar or laser radars and computer vision. ONS systems, meanwhile, are almost exclusively based on GPS (Loomis et al. 2005; Marston et al. 2006). Mobility and navigation assistance have been important research and development topics since 70 s; both are key to the user’s independence and integration within society (Bhowmick and Hazarika 2017). Today, VIPs may own a smartphone and call on a variety of assistive apps to supplement their needs, including BlindSquare, RightHear, and Be My Eyes (Avila et al. 2016; Be My Eyes n.d.; BlindSquare n.d.; RightHear n.d.).

Early navigation assistance products were designed for obstacle avoidance using sonar and laser technology. The user interface (UI) for these devices was typically based on vibrations or sounds (Benjamin 1974; National Research Council 1986). During the 1980s, developers were able to add computational capabilities to these devices. This expanded the range of relayed assistive information by filtering and processing input data, while also improving UI (Maude et al. 1983). The arrival of global navigation satellite systems (GNSS), particularly the global positioning system (GPS) in 1996, made it possible to develop products that allowed VIPs to navigate more independently. These advancements introduced a host of products to the market, such as Trekker, BrailleNote GPS, and Drishti, and signaled that location-based technology had become the backbone of navigation assistance (Library of Congress n.d.; Humanware n.d.; Ponchillia et al. 2007; Helal et al. 2001).

The MOBIC project was one of the earliest research projects for verbal remote navigation assistance aimed at VIPs. It used information from geographical information systems (GIS) and GPS (Petrie et al. 1997). Garaj et al. (2003) then developed a remote navigation assistance system in which a remotely located helper provided real-time navigation assistance using a personal computer. This was accomplished by transmitting a video feed from a digital camera carried by a VIP along with GPS and GIS location information. In turn, Bujacz et al. (2008) developed a prototype where a remotely located helper provided navigation assistance using a video stream from a USB-based camera between two portable computers with wireless internet connectivity. Their prototype was tested in a controlled indoor setting. A more advanced version of this system was developed by Baranski and Strumillo in 2015 and evaluated in a real-world setting. Their results showed the ineffectiveness of the system at busy crossings without traffic lights, as well as low video quality in daylight. Later, they developed an improved version that supported two-way communication, recommending the addition of proper support staff with specialist training as a means to reduce stigmatization and increase acceptance among VIPs (Baranski and Strumillo 2015).

Research has shown that many VIPs are willing to collaborate and communicate with other people when completing navigation- or orientation-related tasks (Balata et al. 2012, 2014). Several commercial applications that provide access to sighted guides are now available. Be My Eyes is a good example of a volunteer community-based AT in widespread use among VIPs (Be My Eyes n.d.). Using the free app, a VIP can establish a video and voice connection to a host of volunteers via their smartphone. The volunteers can assess the situation from the real-time video feed and answer any questions that the VIP might have (Be My Eyes n.d.). However, despite the many benefits of using volunteers (such as real-time assistance and reduced operational costs), quality assurance is a major issue (Avila et al. 2016). In response, the collaborative assistance platform Aira provided communication training to their human agents (Aira n.d.). VizWiz (n.d.), TapTapSee (n.d.), and BeSpecular (n.d.) are other examples of commercial platforms that have incorporated the use of volunteers in a flexible and cost-effective manner.

Traditionally, a number of significant barriers have inhibited the acceptance and adoption of navigation assistance devices among VIPs. Products have been cumbersome, unreliable, expensive, and not widely available (Roentgen et al. 2008; Kim and Cho 2013). Ongoing miniaturization, maturity, and rapid advancements in technology, availability, and cost reduction have since removed many of these barriers (Cardillo and Caddemi, 2019). Another issue for adoption is that the majority of VIPs are elderly. According to Ojamo (2018), an estimated 69% of VIPs in Finland are 65 years old or older, and 57% are 75 or older. They often have other cognitive and physical disabilities, meaning they may need multiple types of AT, and many in the market are not very adaptable. Older people are also slow to adopt new technologies. However, the emerging young elderly group has shown higher adoption rates (Mostaghel 2016). Likewise, younger people with disabilities are more willing to experiment with AT: They are often very goal and task orientated, seeking to enhance their independence and performance (Ripat and Woodgate 2017). Crucially, Roentgen et al. (2012) have highlighted that individual needs and preferences are important determining factors in the acceptance and adoption of ETAs over time. Other factors that can act as either facilitators or barriers include goals, expectations, requirements, functions, functionalities, features of the device, and environment (Roentgen et al. 2012).

## Research methodology

The purpose of this study was to investigate how information and communication technology (ICT)-based navigation assistance can help VIPs and their caretakers in everyday life. Our focus was to study how the acceptance and use of such technology could be increased. We also wanted to know which characteristics of the physical environment need to be considered when developing such a system for VIPs and their caretakers. To answer these research questions, we used a design science research methodology (Hevner et al. 2004; Hevner 2007) to develop the ‘Teleguidance Navigation Assistance System’ artifact. We also needed to evaluate the artifact comprehensively. This led us to investigate how the usability and user experience (UX) of a cooperative AT for VIPs should be evaluated in a field setting. The methods and the setting are presented and discussed in this paper. Originally, we had planned to conduct more extensive studies in different countries and settings; unfortunately, the COVID-19 pandemic prevented us from organizing these additional experiments. However, we have presented the expanded evaluation model based on our plans.

### Target system

**Fig. 1:**
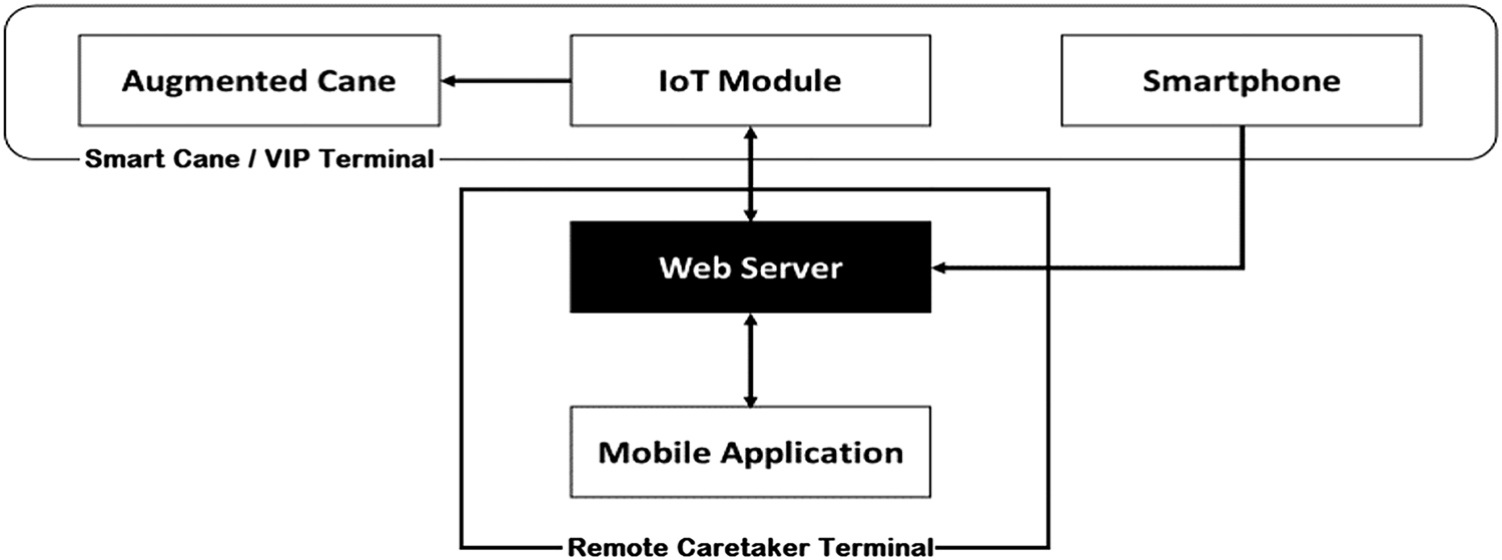
Block diagram of the teleguidance-based navigation assistance system for VIPs

The developed Teleguidance Navigation Assistance system relies on cooperation between VIP and caretaker. It contains a multimodal interface that works over the Internet of things (IoT) and relies on haptic and vocal communication. The system comprises a Smart Cane, Mobile Application, and Web Server (Fig. 1). VIP uses the Smart Cane, composed of an augmented cane, a smartphone, and an open ear Bluetooth earpiece. The caretaker uses a large-screen smartphone with the TeleNavigation App installed. The user interface of the TeleNavigation app shows the VIP’s field of view transmitted from the smartphone placed on the chest of the VIP, along with buttons to control the haptic vibration for VIP guidance.

The system comprises of two terminals:

1. VIP Terminal.
2. Remote Caretaker Terminal.

#### VIP Terminal

The VIP Terminal is a Smart Cane comprised of four components: (a) Augmented Cane, (b) IoT Module, (c) Smartphone, and (d) Open Ear/Bone Conduction Earpiece.

**Fig. 2:**
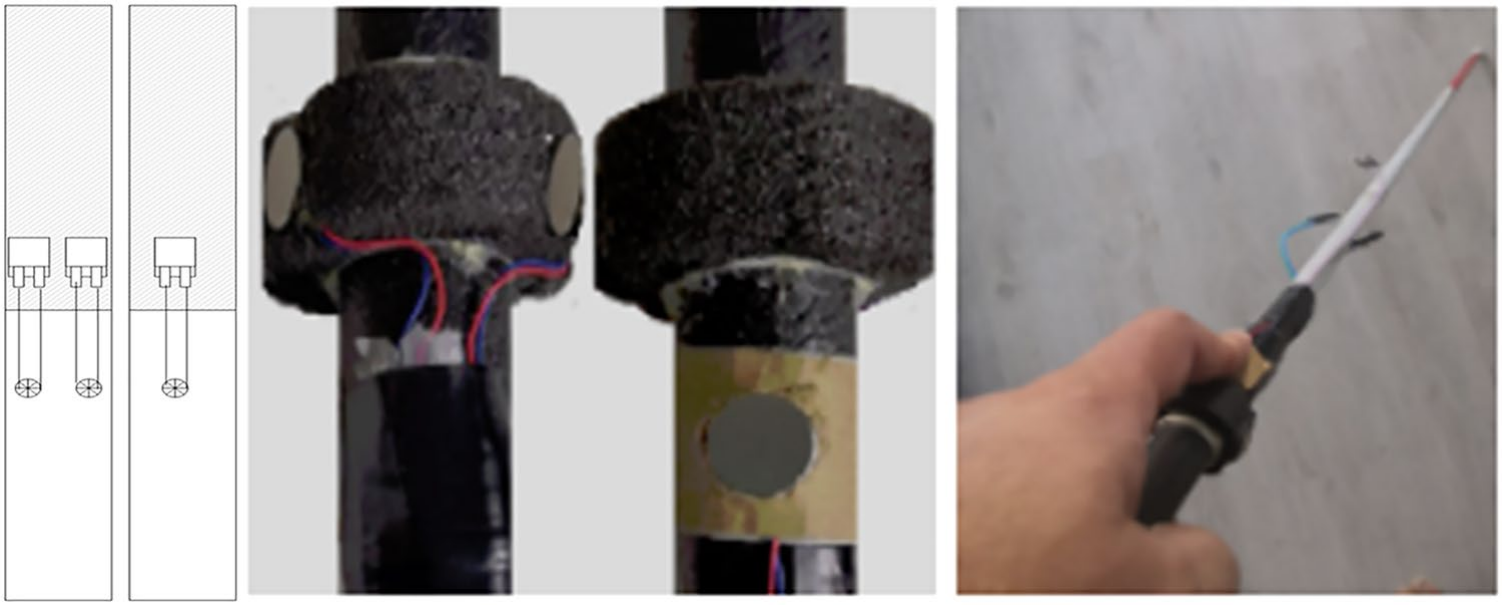
Augmented cane

**Fig. 3:**
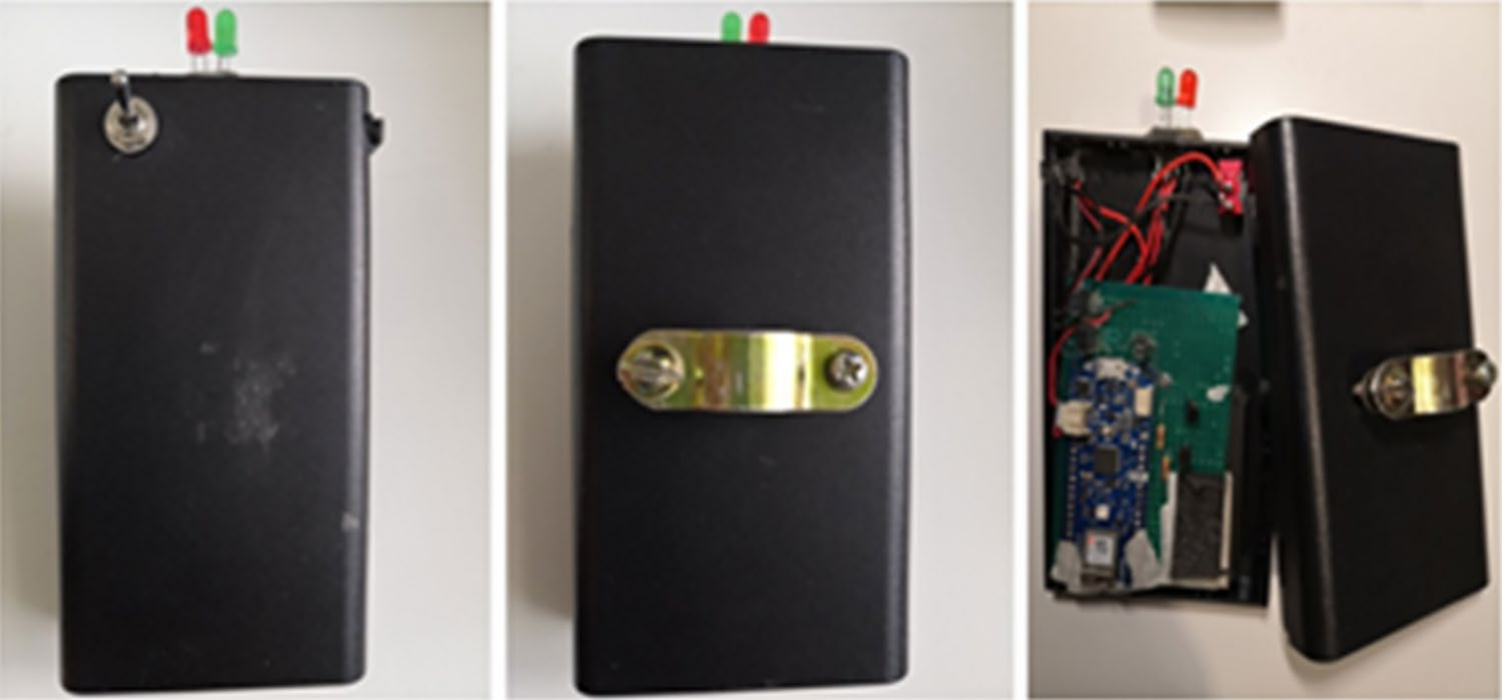
IoT module

- The Augmented Cane is an ordinary white cane with an enhanced grip. The grip includes a tactile UI comprising two alternate haptic methods to relay vibrational navigation instructions (Fig. 2). The first method has two adjacent actuators installed in parallel. The second method utilizes one actuator. These UI elements are installed on opposing sides of the grip. With the two adjacent actuators, the user needs to move either left or right based on which side the actuator is activated. When using one actuator, patterns are used for direction guidance. If the actuator vibrates briefly once, the user should move to the right; if it vibrates three times in quick succession, the user should move left. These patterns match those used by Apple Watch for map directions, allowing us to compare the navigation performance of our system with that of Apple Watch [Apple Watch]. The cane also has open connections for attachment to the IoT Module to receive a vibration control relay from a remote caretaker. The Augmented Cane was developed using a modular approach, so it may also connect to other sources of input. We optimized the position of the actuators so that the VIP could feel the vibrations when using a typical hand grip. This was based on an earlier user study conducted as part of this project [Chaudary et al. 2021]*.*
  The IoT Module is a custom-made removable module that attaches to the Augmented Cane (Fig. 3). It connects to the tactile UI through a wired connection. The circuitry of the chip is controlled by an Arduino MKR1010 MCU [Arduino MKR 1010]. It has Wi-Fi and BLE (Bluetooth Low Energy) connectivity. Connection to the web server is established through Wi-Fi [BLE].
  The Smartphone is hung around the neck of the VIP (via necklet) to send a real-time video feed of the field of view to the caretaker.
- Open Ear or Bone Conduction Bluetooth Earpiece is worn by the VIP in one ear for voice communication with the caretaker.

#### Remote caretaker terminal

The remote caretaker’s terminal comprises two components: (a) Mobile Application and (b) Web Server.

**Fig. 4:**
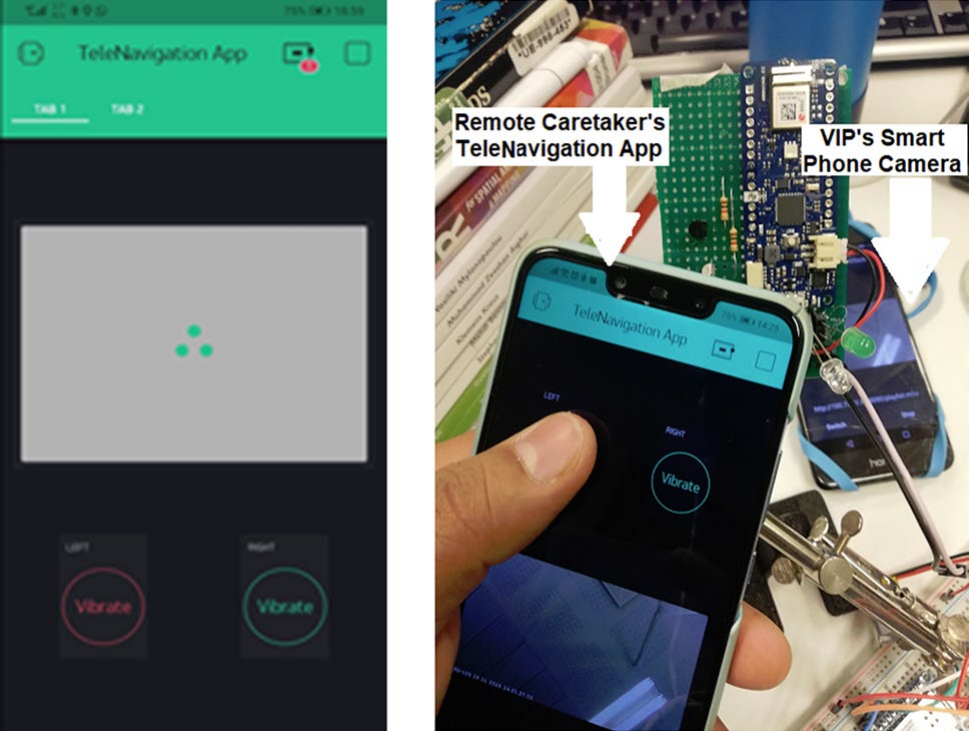
User interface of mobile application (TeleNavigation) for remotely located caretaker

- The Mobile Application is a mobile app developed using the Blynk IoT library installed on the smartphone of the remotely located caretaker [Blynk] (Fig. 4). The UI has a top window that shows a live video feed of the VIP field of view, along with two buttons at the base of the screen to control the actuators on the tactile UI of the Smart Cane.
- The Web Server hosts the control logic of the communication between the Mobile Application and the IoT Module. It is a cloud-hosted development server provided as a service by the Blynk IoT library.

### meCUE 2.0 Questionnaire

ISO 9241–210 (ISO 2010) provides informative formal definitions for both usability and UX. It defines usability as a measure of the effectiveness and efficiency that users obtain from a product, as well as the satisfaction they feel about it. More broadly, UX is an umbrella term used to define many concepts that can also include usability (Majrashi et al. 2015). It also considers user perceptions and responses from both the use and anticipated use of a product (ISO 2010). UX includes emotions, beliefs, preferences, perceptions, behaviors, and psychological and physical responses. These can occur before, during, and after the use of a product. During an interaction, UX is also affected by several other factors, such as brand image, presentation, functionality, performance, interactive behavior, and assistive capability, as well as prior experience, attitudes, personality, and the context in which the interaction takes place (ISO 2010). Elsewhere, Bevan (2009) has explained that different interpretations of UX can lead to different scopes and measures, adding that these can also lead to different concerns. Vermeeren et al. (2010) have argued that usability and UX are closely linked, that UX subsumes usability, and that UX evaluation methods should be augmented with the usability dimension. Arhippainen (2009) sees usability as an interactive experience: It is surrounded by different contexts that contribute to UX at the point of interaction.

In turn, there are many usability and UX methods in the market to evaluate various products from different angles (Chung and Sahari 2015). Some methods are free to use, while others require payment. Over the last decade, several standardized UX questionnaires have emerged, benefitting from user-friendliness, cost-effectiveness, reliability, and research validity. AttrakDiff, UEQ, and meCUE are the most used questionnaires in current academic research. Díaz-Oreiro et al. (2019) have provided a systematic literature review for these standardized questionnaires, noting that AttrakDiff is the most popular by far since it was first to market in 2003, although UEQ has outperformed it in 2017 and 2018. On the other hand, meCUE remains a relative newcomer. According to Díaz-Oreiro et al. (2019), these methods are typically complemented with others. They report that, in over 60% of cases, between one and five additional methods were used. For example, the System Usability Scale (SUS) is a standardized usability questionnaire introduced by Brooke in 1996 and often used in both academic and commercial settings. Importantly, while both AttrakDiff (Hassenzahl et al. 2003) and meCUE (Minge et al. 2017) measure the hedonic and pragmatic dimensions of UX, meCUE also considers the emotional dimension, providing a wider perspective on acceptance-related concerns.

The founding purpose of meCUE was to create a single standardized questionnaire that would address all the key components of UX (Minge et al. 2017). meCUE takes a modular approach to the standardized UX questionnaire, thus allowing for a greater degree of customization. The meCUE questionnaire is in its second incarnation and has five modules that focus on different UX dimensions. The first two modules (I and II) focus on the perception of instrumental and non-instrumental qualities. The third module (III) considers emotions, and the fourth module (IV) focuses on the consequences of use. The fifth module (V) is used for the overall assessment. While the questionnaire is comprehensive, it still overlooks certain aspects of UX, including perceptions of acoustic and haptic quality, and the trustworthiness of the system and the received information. Equally, the questionnaire focuses on purely technical interaction rather than the interpersonal relations or social influences of UX (Minge and Thüring 2018).

Our review of the three standardized UX questionnaires led to the selection of meCUE 2.0 as the research method for this study. At the time, it represented the most current and the most comprehensive method, developed with the most recent research in mind (Minge and Thüring 2018). It was also the only method that incorporated emotions into the evaluation. Besides, this was also an opportunity to test this relatively new method in this particular setting and see how well it performed.

The aim of the study was to validate the system design, but also to investigate the two haptic-based guidance schemes to gauge which the participants preferred and to analyze caretaker feedback to get a better understanding of system UX. The UX-related data were collected through the meCUE 2.0 questionnaire, and a seven-point Likert scale was used (7 = Strongly agree, 6 = Agree, 5 = Somewhat agree, 4 = Neither agree nor disagree, 3 = Somewhat disagree, 2 = Disagree, 1 = Strongly disagree). Conversely, the question on overall experience used a scale from − 5 to 5. The data for usability testing evaluation were gathered from recorded navigation videos, audio recordings of post-scenario questions, and cumulative participant feedback about the system, which included a group discussion. Observations made when familiarizing participants with the system were also an important part of the evaluation process.

### Participants and experiment setup

The usability and UX evaluations of the proposed system were conducted with 11 VIP participants. Their mean age was 32, with a median of 35. Participants comprised three women and eight men. They were recruited through direct contact with two third-party blind associations in Pakistan, Pakistan Foundation fighting Blindness [PFFB] and Prevention of Blindness Trust [POB]. The links to both associations had already been established in earlier studies. An informed consent form was collected from all participants; all participation was voluntary. The caretaker was a 38-year-old sighted female who has worked in the OASIS research group.

Table 1 categorizes the test participants based on visual impairment, gender, and age.

**Table 1:** VIP participants by visual impairment onset/category, gender, and age

Participants	ID1	ID2	ID3	ID4	ID5	ID6	ID7	ID8	ID9	ID10	ID11
Onset	C	C	L	L	C	C	C	L	L	L	C
Category	B1	B2	B1	B2	B1	B2	B1	B2	B2	B2	B1
Gender	F	F	F	M	M	M	M	M	M	M	M
Age	21	21	23	47	36	23	38	44	35	29	44

Onset refers to onset of impairment (C = congenital; L = late). Category refers to the category of visual impairment (B1 = no light perception; B2 = ability to recognize the shape of a hand [USAB])

VIPs used the Augmented Cane, and a Huawei Honor 8 smartphone was hung with a necklet around their neck in the experiment and a Bluetooth open ear earpiece placed in one ear for verbal instructions. The caretaker used a large-screen (6.3″) Galaxy Note 10 smartphone as the remote terminal. Consistent network availability was achieved with a portable Huawei 4G Wi-Fi router. All VIP participants received an oral introduction to the system and the experiment procedure before the test. They were also given the opportunity to handle the Augmented Cane to get a feel for it by touching and grasping. Any questions they had about the system were also answered.

The session started with a test run of the system, which took approximately five minutes. To familiarize the VIP with the system and check the equipment, several tactile cues and voice commands were issued to the VIP. An important protocol to convey was how the VIP should react when taking a turn using vibrational cues. In the case of UI with two actuators, participants needed to turn for as long as they felt the vibration. In the case of a single actuator, when the VIP received a one-time vibrational cue, they needed to turn 45 degrees and then wait (Table 2); if they received no further vibrational cues, they could start walking straight ahead. For voice-based guidance, meanwhile, our earlier research had indicated that VIPs follow instruction more clearly if very simple “turn left/turn right” commands were given; adding further detail only increases mental burden. Therefore, in this experiment, a very simple instruction set was used, as shown in Table 2.

**Table 2:** Haptic and voice guidance methods

Guidance method	Right turn	Left turn
Two vibration actuators	Vibrates until VIP is oriented with new direction after turn	Vibrates until VIP is oriented with new direction after turn
One vibration actuator	A continuous vibration for three seconds	Vibrates three times for one second each with a one-second pause in between
Voice	Turn right	Turn left

**Fig. 5:**
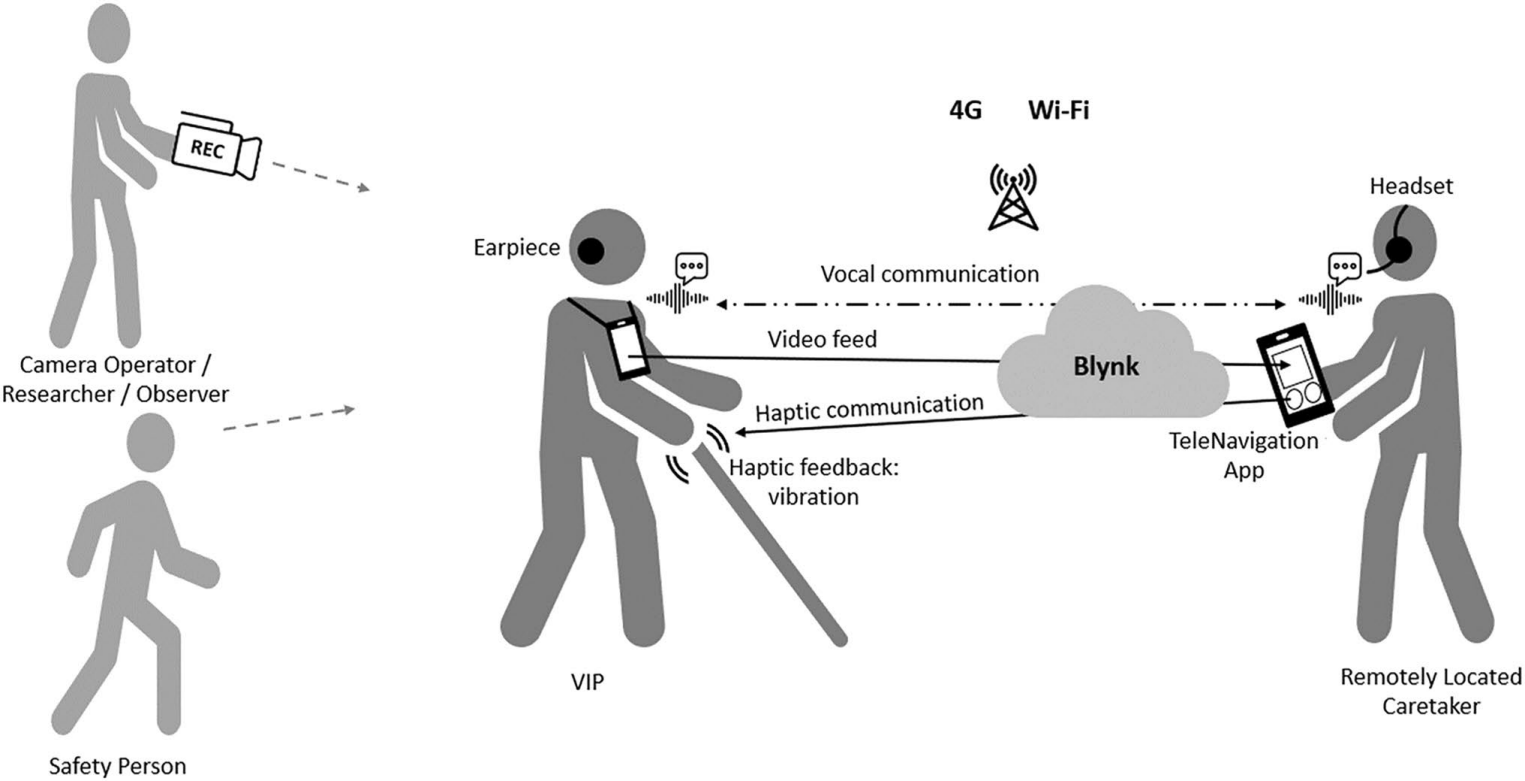
Navigation experiment setting

Figure 5 shows the navigation experiment setting used over three sessions (one indoors and two outdoors). Each session included four participants: VIP, Remotely Located Caretaker, Camera Operator, and Safety Person. The Safety Person monitored the VIP. Each session lasted around 45 min. The navigation took approximately 5–7 min. This was followed by usability feedback questions about navigation through the system and an interview together with the meCUE 2.0 questionnaire and its 34 stated questions. Since the test session with each participant included navigation with both vibration actuators schemes successively for comparison purposes, the system feedback questions were asked at the completion of each route. The meCUE 2.0 questions were then asked at the end of the session.

Tactile communication was the primary method used with the navigation assistance system. Voice commands were used as a secondary navigation assistance method only when needed. This was to limit engaging the auditory senses of the VIP during navigation. The caretaker used voice commands in three situations: If they felt that the VIP had not received a tactile command due to technical error; if they thought that the VIP had misread the cue; or if there was an immediate change in terrain. At the start of the test, the caretaker described the field of view to the VIP. The VIP then asked for permission from the caretaker to start navigating. The caretaker sent the tactile directional cues through the vibration actuator on the Smart Cane to the VIP. The cues directed the VIP at decision points or if they became disoriented. As mentioned above, the caretaker communicated any change of terrain using voice commands, for example, when a surface inclined upward at the start of a paved path.

**Fig. 6:**
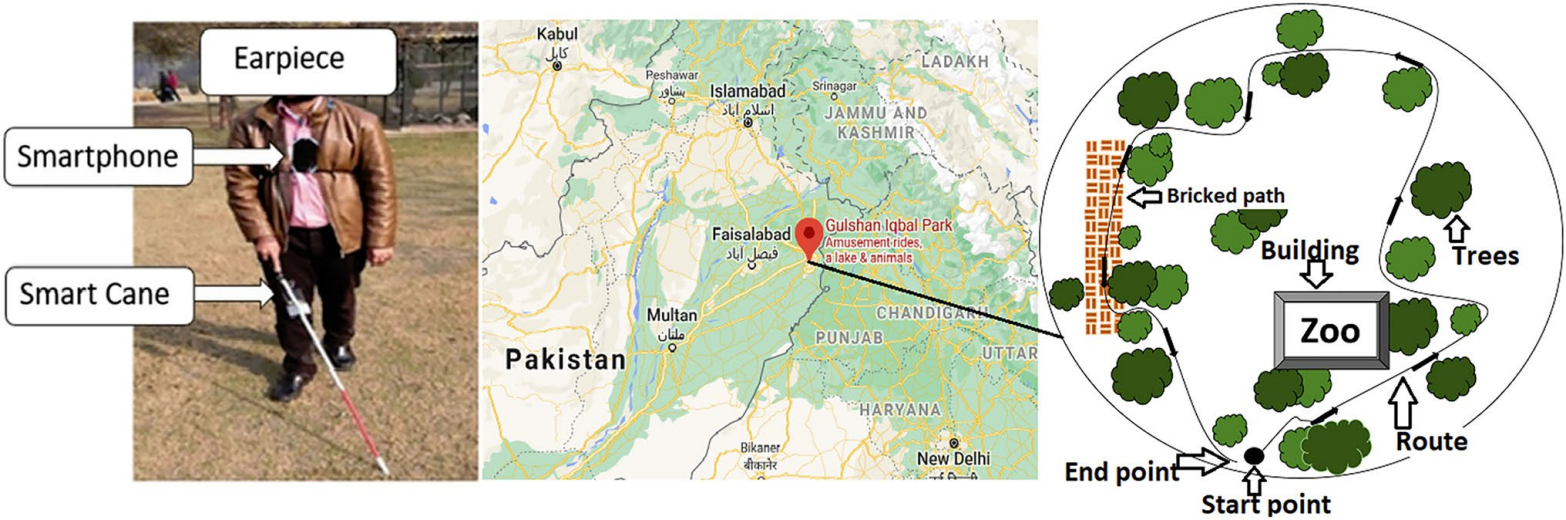
Outdoor experiment setting and navigation task (Gulshan-e-Iqbal Park, Lahore)

**Fig. 7:**
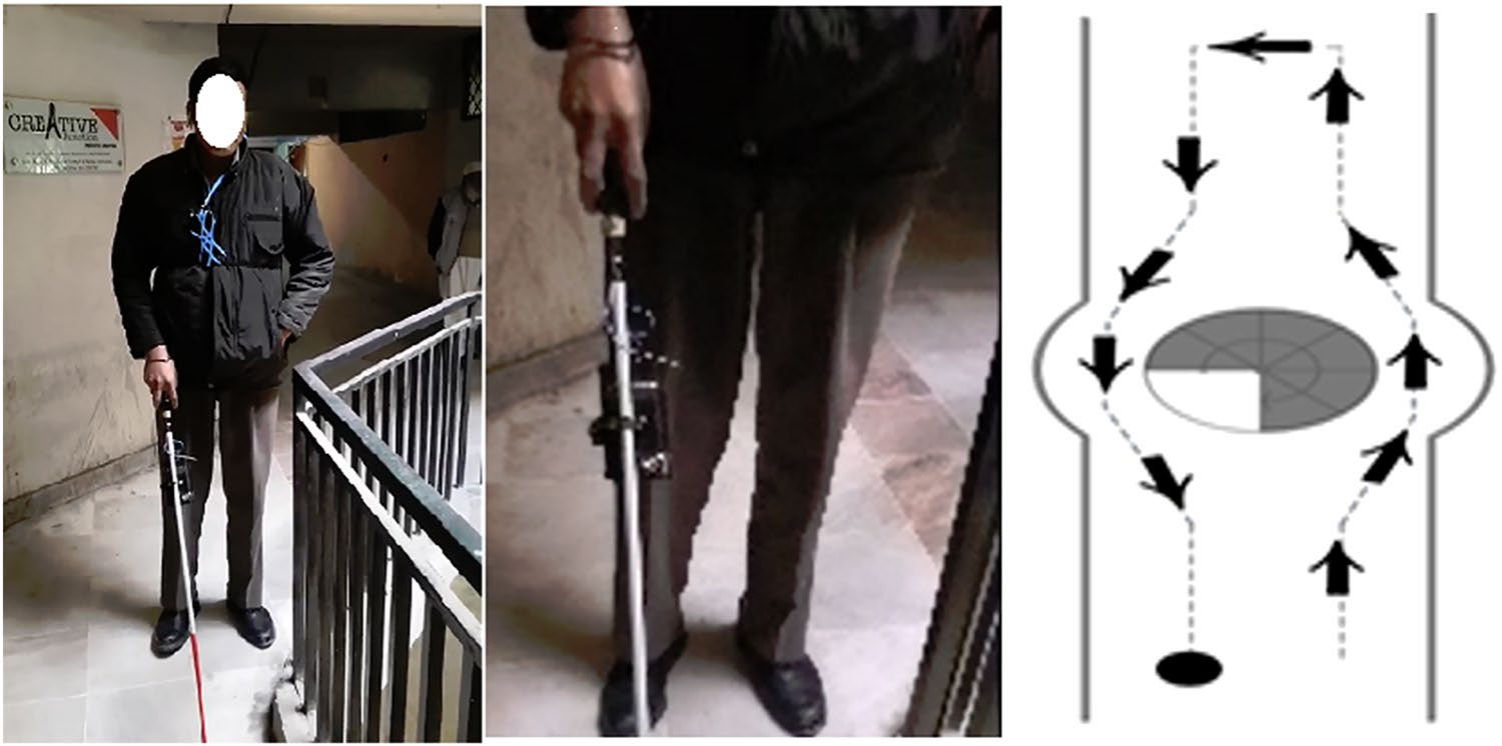
Indoor experiment setting and navigation task (Islamabad)

The experiment took place in indoor (Islamabad) and outdoor (Lahore) environments. The outdoor testing ground was Gulshan-e-Iqbal Park, a public open space that facilitated testing with low levels of interference and hazard (Fig. 6). The indoor route was a corridor in a building (Fig. 7), which contained exit railings and stairways that the participants needed to avoid during the navigation task. The outdoor route had eight decision points and changes of terrain; the indoor route had ten decision points (Fig. 7). The length of the outdoor route was about 250 feet (76 m), while the indoor route measured about 150 feet (46 m).

Each navigation scenario was tested using both haptic vibration schemes. Data gathering was based on field laboratory design recommendations by Høegh et al. (2008). All tests were video recorded for analysis. At the completion of each scenario, post-scenario questions were asked and their responses were audio recorded. Cumulative feedback on system usability and acceptance was requested and audio recorded at the end of the test. Interviews about UX were then conducted using the meCUE 2.0 questionnaire; responses were marked on a paper sheet. All communication with the participants was in their native language (Urdu). Their quotations were subsequently translated into English.

## Data analysis and findings

This section examines the user experiences after the VIP participants had completed the navigation tasks by following cues from the remote caretaker. Both haptic navigation methods offered by the system were tested. The UX data were analyzed using the meCUE 2.0 questionnaire template (meCUE 2.0, n.d.).

### meCUE 2.0 questionnaire findings

**Fig. 8:**
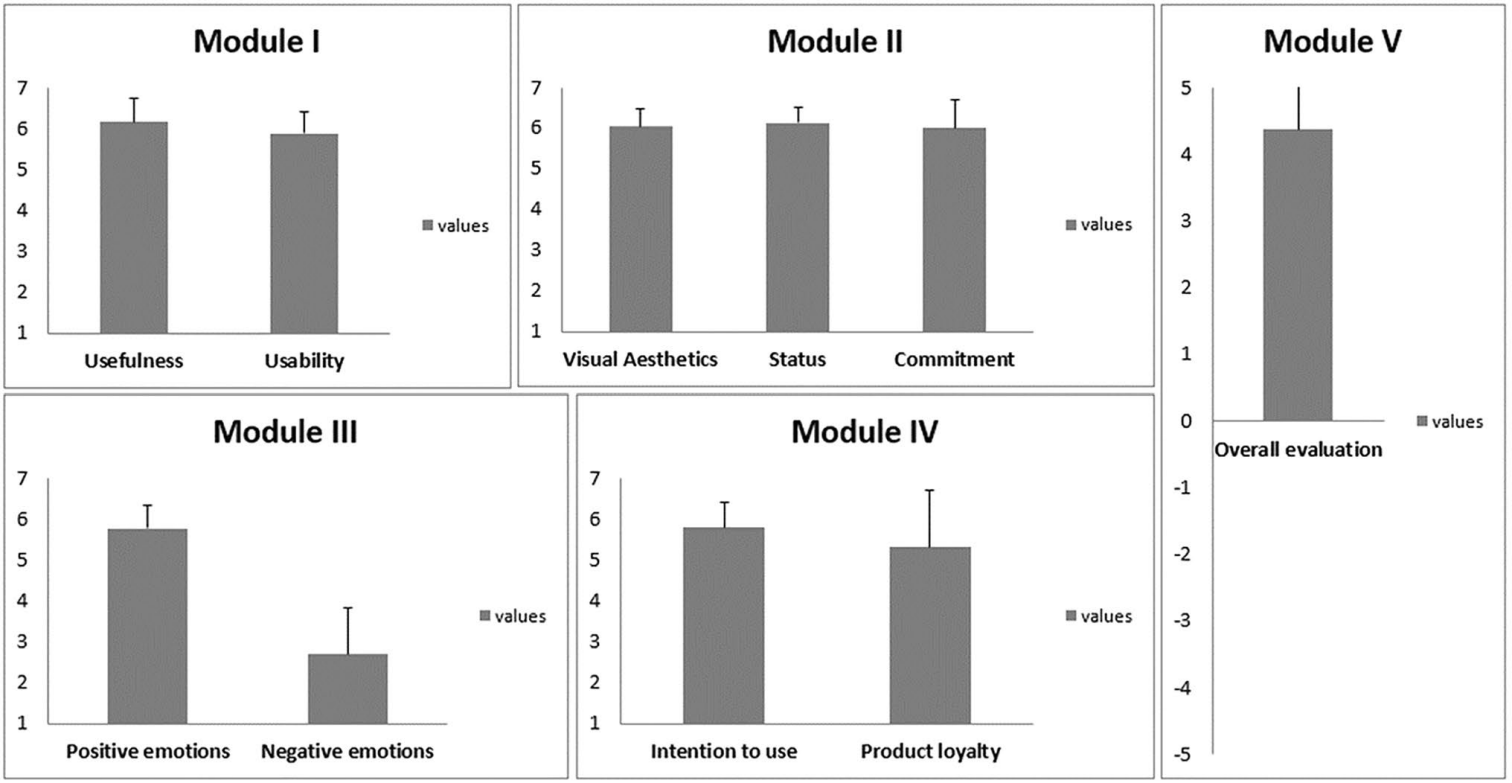
Module mean values and standard deviations according to VIP meCUE 2.0 questionnaires

Our findings (Fig. 8) show that feedback on the system was generally positive. Participants perceived the system as both usable and useful (Module I). Two participants (ID8, ID11) regarded the system as particularly useful for navigation: As ID11 commented, “My sister, who usually has to help me navigate by accompanying me, could assist me from home with this [system].”

Based on participant feedback, our system has the potential to increase the independent mobility of VIPs. One participant commented that the system could help them connect with family and get remote assistance without the need for physical contact. Another participant gave a good example of a possible use case when there is a need for help from strangers:

You cannot get help from anyone anytime but using this [system] it could be possible. If I go somewhere and need help, I must ask someone for help. If he/she refuses for some reason, it makes me feel bad: why am I in such a situation that I had to ask for help? That’s why I find this system very good. With its help, I could receive support from my caretaker and travel easily (ID1).

According to the meCUE 2.0 questionnaire results (Fig. 8), visual aesthetics, status, and commitment (Module II) were also appreciated. This is a good result, especially since the system was in a prototype development phase. In comparison to the regular white cane, participants felt that this type of system could be more useful because of the extra aid it provides. More specifically, the regular cane was perceived to be more limited (ID4) and lacking in informational feedback (ID2). However, participants also expressed concern about the technical aspects of the new system; for example, “Sometimes the connection is not good, which creates a problem. There needs to be some work done on that” (ID7) and “The connection lost problem should be considered too” (ID5). Furthermore, as another participant commented:

The position of the vibration actuators is not optimal for grip handling. It would be better if the actuator were in a rectangular shape than a rounded shape. It would be better to use a separate wirelessly connected camera, which is movable so the remote caretaker can look around, rather than the mobile phone’s camera (ID10).

Another participant elaborated on the camera view issue as follows:

But the front-facing view on its own is not enough for the remote caretaker. He should be able to see sideways and backwards, too. So, he could select the view from his device if he needed to see the other sides. A camera other than a mobile phone would be better. As if I need to take a call, it would be difficult to set up [the system] again (ID5).

Lastly, some participants thought more practice would be needed to become proficient with the system. One participant proposed an improvement idea: “If an autonomous function could be added that records a route like on a map one time and we could use it later without help from remote assistance to reach that destination, that would be very beneficial” (ID5).

Emotions (Module III) were mainly positive (Fig. 8). Two participants (ID1, ID10) experienced negative emotions. They felt that the system made them tired, as using it was exhausting. One participant (ID11) stated that the system made him feel passive. Positive emotions related to possibilities that the system could provide to the lives of VIPs: Statements included “it would bring a happy change to the lives of blind people” (ID4) and “it can change our lives” (ID2). In addition, one participant reported a greater sense of personal security: “Now when we have to be on our own, by using [the system] we are informed beforehand about approaching situations. That will increase our security” (ID3).

Intention to use and product loyalty (Module IV) were rated reasonably highly (Fig. 8). For instance, all participants thought that they would use this system daily. Also, 64% of participants answered that they would not swap this product for any other. The overall evaluation (Module V) of the system received high marks with a mean value of 4.4 (on a scale of -5 to 5). Guidance was particularly highlighted as a system benefit:

We can follow the directional guidance well using this system. It can change our lives. It will be easy to reach our destination. Now we use a normal cane, but that does not provide much information. But if someone guides us toward our destination, it will make it very easy (ID2).

**Fig. 9:**
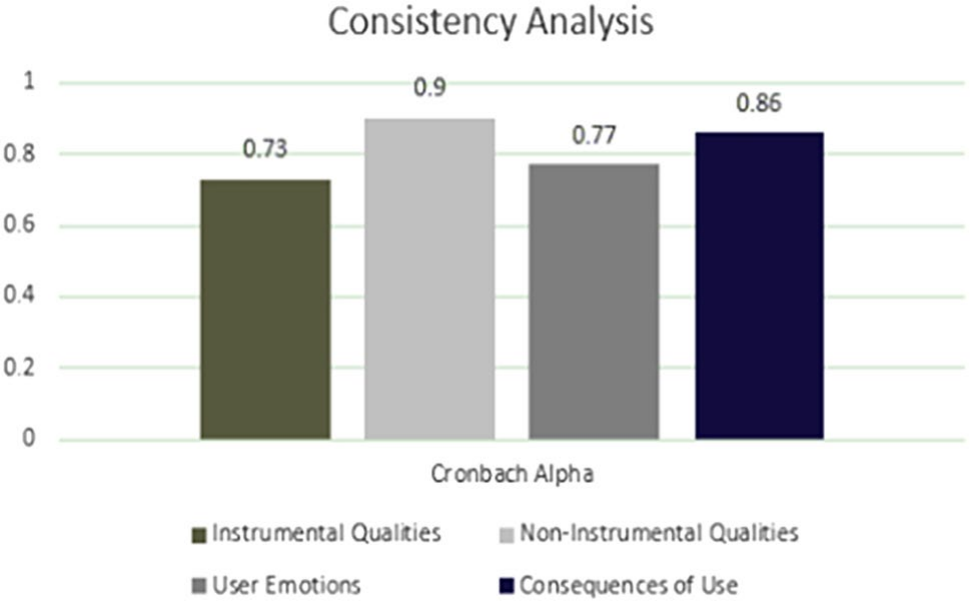
Consistency analysis results for meCUE 2.0 questionnaire responses

The Cronbach’s alpha test was used to check the consistency of the response data (Fig. 9). All values for this reliability test ranged from 0.73 to 0.86; the general rule of thumb is that a Cronbach’s alpha score of 0.70 or above is good. This suggests that all indicators measured by the questions in this research are reliable.

### Findings based on background questionnaire, interviews, and group discussions

Video recordings, interviews, and background data were analyzed as summarized in Table 4. UI scheme preference was categorized into four groups (G1–G4) that shared similar user characteristics.

The results show that the default choice of blind participants (G1) is the two-actuator setup if they are 29 years old or above, while the one-actuator system was favored by those who were VIP and aged 29 or above (G3). Younger VIPs (up to 23 years old), despite their visual acuity, preferred the two-actuator system (G4). For blind participants who were not regular cane users and who were aged 29 or above, the default choice was one actuator (G2). Following observations and discussions with participants, this behavior was rationalized. The blind participants had their full concentration on the Smart Cane when navigating: It was therefore easier for them to understand instructions from the twin actuators. A few blind participants even suggested adding a third actuator that would give them haptic cues about when to stop or start walking again. On the other hand, the visually impaired participants (with some limited vision remaining) reported that using two actuators made them focus too much on the cane; it proved easier for them to glean instruction from one actuator, as the mental load was lower. This behavior was associated with regular white cane users. If a participant was not a regular user of a white cane or had never used one before, then age became a factor.

Further study is required with younger users who are under 24. Here, younger users, whether fully blind or VIP, seemed to prefer two actuators, as they have the capacity to learn the required skill. Older participants, even those with little or no white cane experience, found it easier to learn to use a single-actuator system. Table 3 describes this preference between groups of participants, while Table 4 presents the rationales for their setup preferences. Ultimately, several participants commented that learning to use the single- or twin-actuator setup requires a certain degree of practice. In future studies, it would also be important to conduct long-term evaluations; for instance, participants could use the system for one or two weeks and report their experiences using voice diaries. As we do not have any participants with age between 23 to 29, that remains a gray area. It needs more tests with this age group to find their preferences that might be overlapping in age case.

**Table 3:** Preferences matrix for user interface schemes by participant group (G1–G4)

			User characteristics	Preference for one or two vibration actuator(s)				
G	Blind/VIP		Regular cane user	Age < 24	First preference		Second preference	
1	Blind		Yes	Yes	Two		One	
1	Blind		Yes	No	Two		One	
1	Blind		No	Yes	Two		One	
2	Blind		No	No	One		Two	
3	VIP		Yes	Yes	One		Two	
3	VIP		Yes	No	One		Two	
3	VIP		No	No	One		Two	
4	VIP		No	Yes	Two		One

**Table 4:** Participant rationales for positive and negative aspects of single- and twin-vibration setup

Positive arguments	Single vibration	Usable Easy to follow Can understand the patterns to follow the direction Easy to concentrate on one vibrator for all guidance Possible to follow the left- and right-moving cues by differentiating the patterns
	Twin vibration	Easy to use Possible to follow the directions of the remote guide Easy to follow guidance Easy to differentiate between the two sides and follow guidance accordingly Possible to follow the directional cues from a remote caretaker Very helpful to receive left/right movement guidance through different vibrators Easy to use cane with two fingers aside the grip Convenient to follow
Negative arguments	Single vibration	Understandable but sometimes confusing Difficult to understand because the duration of vibration is short Confusing because vibration patterns are too fast Usable but vibration timing needs to be longer Connection sometimes lost and patterns cause confusion Not very helpful for left/right movement guidance
	Twin vibration	Vibration duration should be longer, so it is not missed if user is inattentive at given time Confusing Requires some practice to learn

### Preliminary findings based on caretaker’s experiences

It is important to investigate the perspectives of caretakers when evaluating a cooperative navigation assistance system. Equally, the role of caretaker should be considered when preparing the experiment procedures, UX methods, and questionnaires. Caretakers have different experiences with the system than VIPs, given their different roles as users. It is worth noting that, in this study, only one person participated in the role of caretaker. This person was also a part of the research team. In our opinion, it is still appropriate to report these preliminary findings, as our aim is to include more caretakers in future studies. These caretakers will have different backgrounds and relationships to the VIPs. They can be professional caretakers, non-professional volunteer caretakers, family members, or friends.

In this study, we were interested in the usability aspects of the caretaker terminal UI as part of the overall system. As illustrated in Table 5, feedback was gathered via discussion between the test participants, the researcher, and the caretaker. It also included analysis of the video recordings from the experiment. These findings are tentative, as we were only able to incorporate a single caretaker into the experiment procedure; this should be taken into account when improving the usability and UX of the assistive system. Based on the existing feedback from VIP participants and the caretaker, certain technical aspects of the system require improvement in order to reduce disconnections, thereby boosting system reliability.

**Table 5:** Usability feedback on caretaker terminal UI and system usage

Indoor environment	Guidance is challenging in buildings where more small objects are around Voice communication is clearer indoors Detecting the environment is easier (e.g., how large or wide a place is, how surroundings change, or how the presence of a crowd is fØelt through echoing sounds) It is easy to judge how well the participants are following the given guidance, i.e., left/right turns relative to surroundings Low radio signal availability is a particularly critical issue indoors, where the margin for error is lower
Outdoor environment	The view of surroundings is clearer because of the more open environment It is easy to re-orient the participants using vibrations, as there is more room to maneuver Low echoes and sideways voices are less indicative of surroundings compared to indoors Audio helps in recovery if video is lost, because the caretaker has a better understanding of the environment
Navigating blind and VIP participants	For blind participants familiar with white cane use, adapting to the system was relatively easy despite age or other factors For blind participants unfamiliar with white cane use, their complete focus was on learning to use the cane and observing caretaker feedback Blind participants responded to haptic cues quicker and made fewer errors in understanding haptic instructions than VIPs Visually impaired participants’ attention was somewhat diverted during navigation as they also tried to use their remaining sight to make sense of the environment Visually impaired participants’ response to haptic assistance was comparatively slower
Switching between haptic and voice commands	Voice guidance was understood quickly Switching back to vibration after voice instruction was slower for participants; it took several seconds for them to start using vibration smoothly again A meticulous and strategic use of voice-based guidance was required
Small smartphone screen	The screen size of the smartphone was found to be sufficient to observe the participants’ field of view [Samsung Galaxy Note 10] The position and angle of the smartphone camera are important
Participant safety	When outdoors, the caretaker can see potential hazards up ahead When indoors, consistent assistance is required, as there is less room for error

## Expanded model for usability and user experience evaluation

Initially, our research plans were to conduct further experiments using this system in Finland, Pakistan, and Sweden. As mentioned above, the experiment in Pakistan only utilized a single caretaker; it is essential to test the system with a greater number of caretakers. This also includes having multiple caretakers use the system at the same time. These experiments were due to start in the second quarter of 2020 but were delayed by COVID-19. Unfortunately, it is also unlikely that the research team will be able to conduct them in the short term, given current social-distancing measures, and particularly since research has shown that elderly people and those with other comorbidities have a higher risk of COVID-19 mortality (Guan et al. 2020; Sanyaolu et al. 2020). The fact is that many VIPs are elderly and belong to this high-risk category. Ultimately, the risks to participants would be too great to conduct any further experiments for the foreseeable future. As a result, in this section, we introduce our expanded evaluation model, process, and procedure—the Usability and User Experience of Cooperative Assistive Technology for Blind and Visually Impaired People (UUXCAT)—with the intention of improving future experiments with similar systems. The UUXCAT model considers the latest research and developments in usability and UX evaluation. It also adds several contexts previously missing from standardized usability and UX evaluation methods, but which are nonetheless significant when developing assistive technologies for VIPs.

**Fig. 10:**
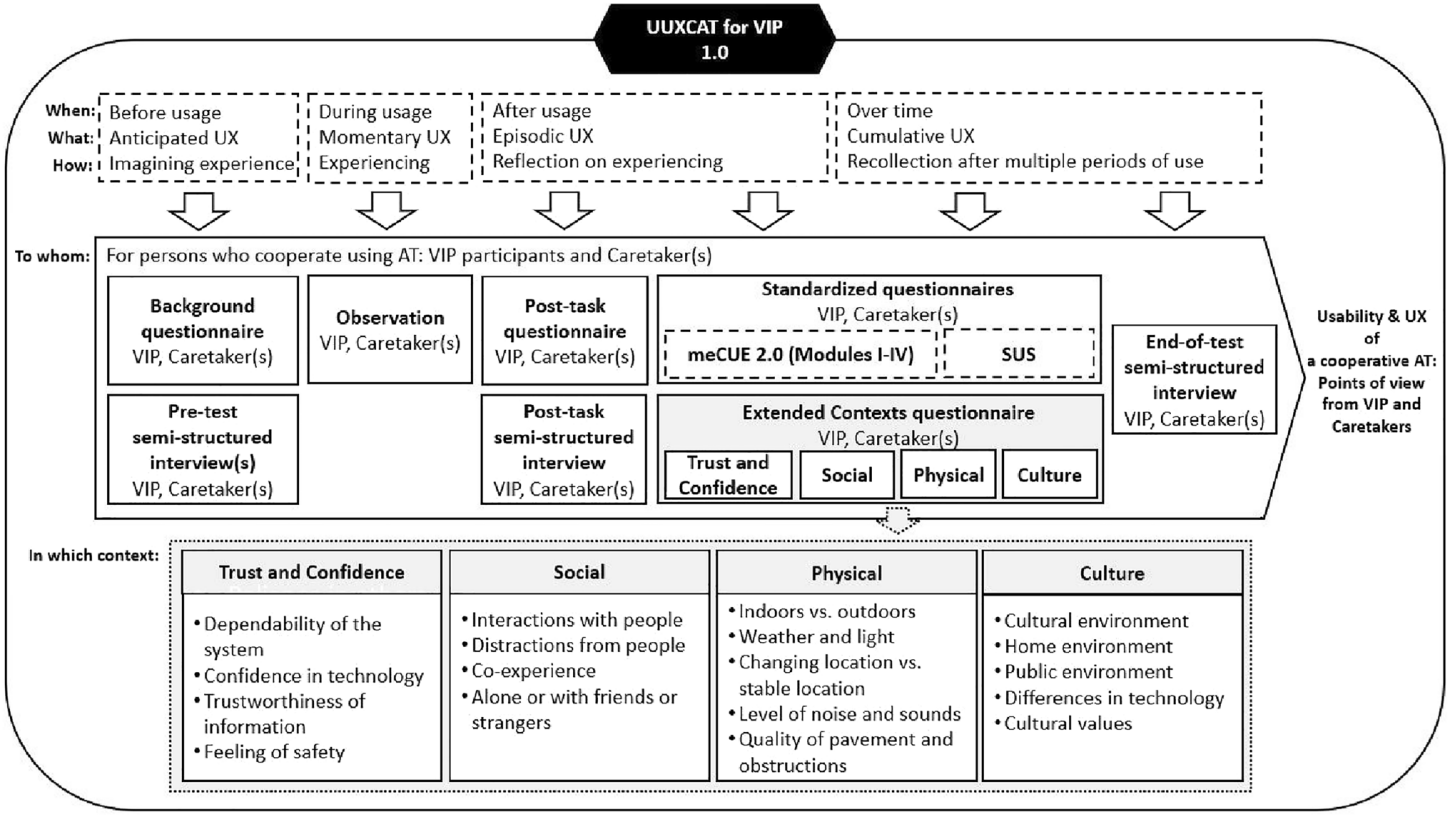
UUXCAT model for VIP evaluation

Figure 10 shows the evaluation model (Pohjolainen, 2020). While our experiments were developed with this model in mind, they are modular and adjustable: They can be customized to suit different settings with a selection of different evaluation methods. Certain methods can differ greatly depending on the length and location of the experiment; for example, the selected methods should reflect whether the experiment takes place in a laboratory, in the field, or in a natural environment. Here, the model shown incorporates the evaluation of VIPs and their caretakers using a cooperative system in a field setting. The research team has conducted an overall assessment of the evaluation model and checked for any systematic errors.

The dotted lines in Fig. 10 indicate the existing work of UX professionals. The periods of experience highlight the need to obtain feedback from participants at the different stages of the evaluation process—as Roto et al. (2011) have explained, the views of participants can change significantly between these periods. The model also displays the methods used to capture a particular usage period. For example, to capture momentary UX during usage, observing the participants was chosen as the least intrusive method in our setting, since it was important that communication between VIP and caretaker was not interrupted during the navigation task. In this case, observation involves not only monitoring the participants, but also collecting system data during usage. The evaluation model incorporates the meCUE 2.0 questionnaire from Minge and Thüring (2018) and the SUS usability questionnaire from Brooke (1996). The latter was selected as it adds greater weight to issues of usability than meCUE 2.0 and has been reliably and extensively used in both academia and industry. In our model, standardized questionnaires capture aspects of both episodic UX (reflection on experience after usage) and cumulative UX (recollection after multiple periods of use).

Crucially, the UUXCAT model incorporates four contexts not covered (either at all or not to any meaningful extent) by existing standardized questionnaires when evaluating this type of cooperative system with VIPs and their caretakers. These contexts are Trust and Confidence, Social, Physical, and Culture. Pohjolainen (2020) has presented the research on these contexts in more detail. These contexts are modular and developed for our experiments. However, they can be either selected or adjusted depending on requirements.

**Fig. 11:**
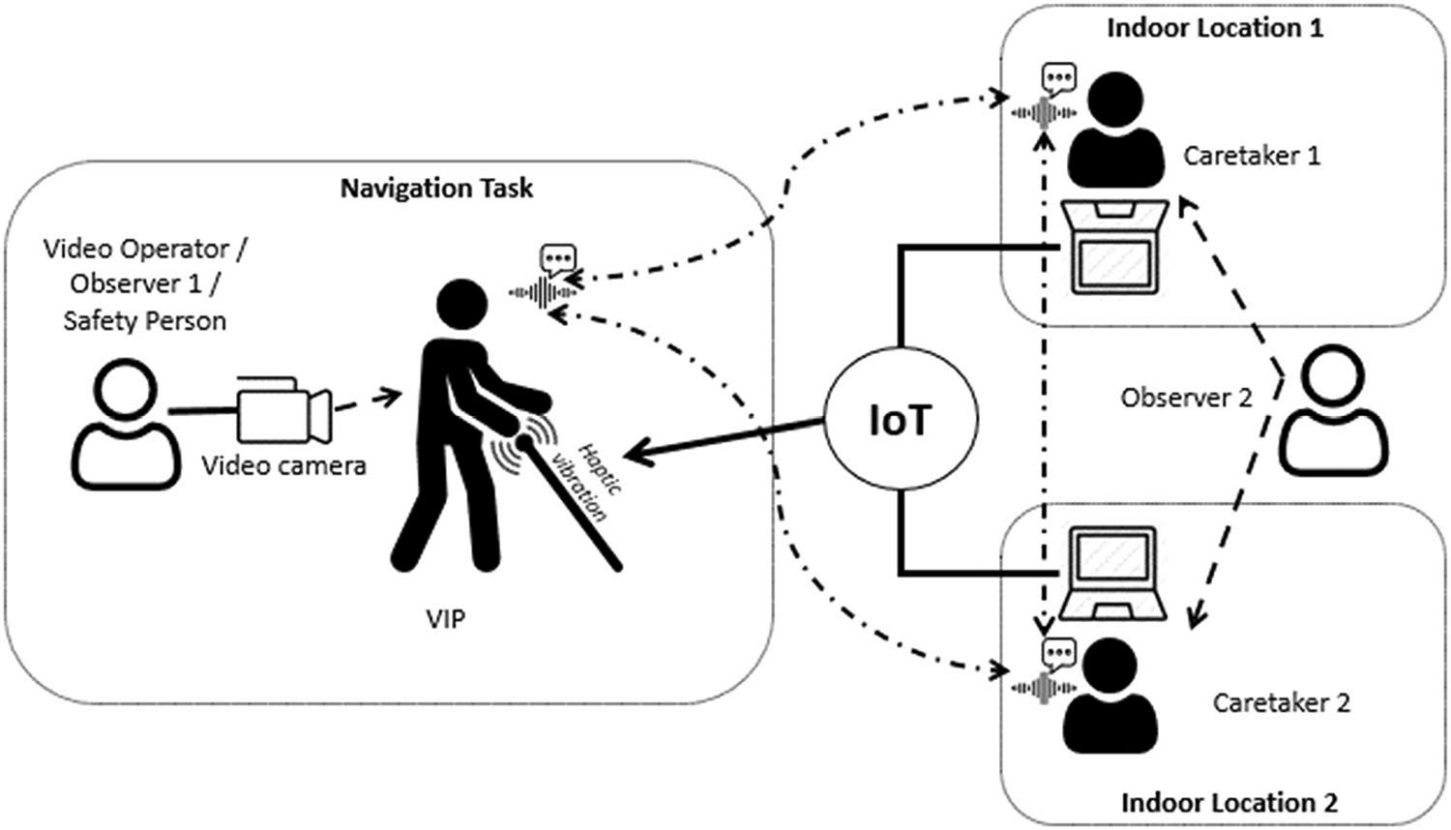
Evaluation setting example with two caretakers

Figure 11 illustrates one of our planned evaluation settings using two caretakers. It is an evolution of the setting presented in Fig. 5. This experiment was to take place during the second quarter of 2020 and include three participants (VIP, caretaker 1, caretaker 2) and two observers per test. The third participant, caretaker 2, is a somewhat specialized role, as they would be familiar with the locale and the system. His/her role would be to support and interact with caretaker 1 and the VIP during certain parts of the navigation tasks. Observer 1 is present to record the navigation tasks and interviews with VIPs on video. Observer 1 also acts as the Safety Person for VIPs when needed. Observer 2 records only voice with caretakers but also makes notes while observing them during the navigation tasks. Two different navigation tasks were planned for each test group.

The evaluation procedure contains five parts, each of which includes tasks associated with the UUXCAT model shown in Fig. 10. The first part concerns completing the background questionnaire along with the consent form. It is also the point at which participants are introduced and receive instruction on the use of the system. In the second part, a short pre-test semi-structured interview is carried out. After the interview, participants are led through an orientation task to become familiar with the system. In the third part, the first navigation task is conducted. After this task, caretakers fill out a short post-task questionnaire, while the VIP answers a few post-task questions. The fourth part mimics the process from the third, but the navigation itself takes place in a different location. A short break follows this part, allowing participants to get refreshments from the local cafeteria: This allows a period of time to pass before asking participants to complete the post-test questionnaires and end-of-test interviews, thereby enhancing cumulative UX responses. The fifth and final part involves the completion of three post-test questionnaires by all participants (meCUE 2.0, SUS, and Context Modules), followed by short end-of-test semi-structured interviews.

## Discussion

In this section, we discuss the limitations of our study and potential future research, in terms of both the developed system and the UUXCAT evaluation model.

## Limitations

We acknowledge that our findings are tentative, and we cannot generalize the results based on the small sample size. Future studies would need to include more VIP participants and caretakers from various backgrounds in order to understand different AT users’ needs and wishes more comprehensively. For instance, we should conduct studies with VIP participants from different age groups (e.g., children, young adults, middle-aged adults, older adults), nationalities, and living environments. Also, the previous experiences of VIPs with the regular white cane and other assistive technologies should be taken into consideration. In addition to descriptive statistics, we considered using nonparametric test for inference statistics, but as the sample size is small, this was not justifiable. For example, the Fisher’s exact test, that would have been otherwise used, is not recommended if more than 50% of the cells have expected count less than 5, and that would be the case for our sample.

The number of caretakers was another major limitation in this study, since only one caretaker participated, and that person belonged to the research team. In future studies, recruiting several professional and non-professional caretakers is of critical importance. The caretakers should also have no ties to the research team. Understanding different types of caretakers in various experimental settings is a worthwhile research direction. For example, instructors or teachers of VIPs who are familiar with assistive technologies could take a caretaker role, while experiment settings could include a work- or leisure-based scenario or a short but intense navigation route. Other tests could utilize friends of VIPs as caretakers, or absolute strangers. As identified by our participants here, there can be crucial differences between asking or receiving help from a stranger, family member, or caretaker; these differences are worthy of further and closer consideration.

Another concern that arose from this experiment was the participant bias issue, which is known to produce a significant amount of error and therefore must be avoided (Dell et al. 2012). While participant bias is a well-understood issue, it is not often considered when carrying out field experiments with VIPs. For example, it may be possible to avoid mentioning that the interviewer was part of the research team (e.g., by saying he/she was conducting the interviews on their behalf). This could at least avoid some participant bias. Otherwise, questionnaires could be presented in Braille, so that VIPs could read the questions themselves.

We acknowledge that further studies are also required from the viewpoint of usability and UX methodology. This was the first time we applied the meCUE 2.0 questionnaire to VIP participants and caretakers. In future research, we should be able to take different contextual aspects into account. For instance, it is important to understand whether VIPs feel that they can safely use the system to navigate from one location to another, or whether caretakers think that the system is dependable enough to help them guide a VIP to their destination. It is also important to study whether the users feel safe using the system and how much confidence they have in the assistive technology. The system should also feel dependable and trustworthy (Schrepp and Thomaschewski 2019), so that VIP does not stumble into obstacles because of delays in communication or turns in the wrong direction when receiving information from the caretaker. The social context should also be considered, as the assistive system relies on cooperation with a caretaker. While these safety and social aspects were missing from the meCUE 2.0 questionnaires, we took them into consideration in the expanded UUXCAT evaluation model.

## Future Work

Based on this study, we have identified several future directions for system development and experiment setting. In addition to the usage aspects outlined above, it is also important to consider the environmental aspect, as the physical user environment can impact use of the system. For example, low pavement quality or the presence of many obstructions can make it difficult for a VIP to traverse the terrain. Noises and other sounds can reduce the user’s attention, while changing weather conditions and seasons can affect UX considerably. Likewise, lighting or temperature levels can make using the system more difficult, which in turn impacts UX (Forlizzi 2008; Nicólas and Aurisicchio 2011). Further field studies, particularly in various real-life situations (such as walking to a shop or meeting up with friends), would thus be beneficial.

The use of technology and the integration of VIPs into society can vary significantly between countries, as can the possibility of using white canes, guide dogs, and adaptive transport services. According to Ripat and Woodgate (2011), some users of AT may identify with a disability culture that contains its own shared set of beliefs, values, and behaviors. While users may have their own habits and rules, it is important to consider the larger view. This includes differences between workplace or organizational cultures, current level and availability of technology, favored products, and general acceptance of products in a given culture or sub-culture (Arhippainen 2009; Nicólas and Aurisicchio 2011). To take these cultural aspects into account during system development, it would thus be beneficial to conduct user studies across different countries.

To cover many of the aforementioned research limitations, we have introduced the UUXCAT model for VIP evaluation. Clearly, there is a continued need to identify methods for capturing those additional contexts and factors that are likely to be important to VIPs and their caretakers. Some of these contexts were mentioned by participants in this study; Pohjolainen (2020) has provided our questionnaire based on these contexts. However, the major limitation of our expanded model is that it has not been validated through testing. In future work, therefore, more modules and factors could also be validated and integrated into the evaluation model.

At the time of writing, there are at least three main areas that should be addressed in future work with the system in development: (a) *conducting a test with a 5G network*; (b) *testing the system with two caretakers*, where a secondary caretaker acts as a mentor in situations that are unfamiliar to the primary caretaker; and (c) *automating and delegating part of the guidance using AI support or prerecorded voice-based commands*. The 5G network tests are critical to assess the performance of the system in crowded spaces, especially during turns. In addition, our usability testing revealed that different participants have varying preferences about the position of the vibrators and the time and frequency of command vibrations. In an updated prototype of the system, these aspects can be adjustable so users can tailor them to their own preferences. Finally, the evaluation model and the added contexts should be validated with a field experiment.

## Conclusions

This paper has outlined the development of a specialized teleguidance-based navigation assistance system and the results of a field experiment conducted with 11 VIPs and one remote sighted caretaker. In the experiment, the VIP sent a live video feed of their field-of-view to the remote caretaker’s terminal via a smartphone camera attached to their chest. The caretaker used this video feed to guide the VIP through different indoor and outdoor navigation scenarios using a combination of haptic and voice-based communication. Haptic-based and voice-based commands were used as the primary and secondary means of communication, respectively. The haptic feedback was provided through vibrating actuators installed in the grip of a Smart Cane. Two alternative haptic methods for directional guidance were tested: twin vibrating actuators and a single vibrating actuator.

Overall, feedback from the participants was positive about the proposed navigation assistance system. According to our findings, it seems that blind participants preferred vibrational guidance with twin actuators, while partially blind participants preferred the single actuator method. Familiarity with cane use and age were also found to be important factors in the choice of haptic methods by both blind and visually impaired user groups. Young adults (up to the age of 24) preferred twin actuators despite their visual acuity level, while blind adults aged 29 and above who were unfamiliar with the white cane preferred a single actuator. Our findings show that VIPs are positive about the navigation assistance offered by the system, but that they also have different preferences. Moreover, the smartphone camera provided adequate field of view for remote navigation assistance, but camera position and angle are important to consider in future development.

This paper contributes to the understanding of the user experiences achievable for blind and visually impaired people with this type of navigation assistance. Though meCue 2.0 was successfully used in this study, we emphasize that more studies are needed in different circumstances and more participants to confirm our preliminary findings. In addition, this paper introduces the expanded evaluation model (UUXCAT), which was developed in preparation for further experiments with the system, but ultimately could not be implemented because of the COVID-19 pandemic. The UUXCAT model includes usability and UX methods to study cooperative navigation assistance systems involving VIPs and their caretakers. It also introduces several new contexts related to VIP users that were previously missing from the standardized usability and UX questionnaires.
